# Beta-Adrenergic Receptor Blockade Effects on Cardio-Pulmonary Exercise Testing in Healthy Young Adults: A Randomized, Placebo-Controlled Trial

**DOI:** 10.1186/s40798-022-00537-5

**Published:** 2022-12-20

**Authors:** Kevin Forton, Michel Lamotte, Alexis Gillet, Martin Chaumont, Philippe van de Borne, Vitalie Faoro

**Affiliations:** 1 Cardio-Pulmonary Exercise Laboratory, Faculty of Motor Sciences, Université Libre de Bruxelles, Erasme Campus CP 604, 808 Lennik Road, 1070 Brussels, Belgium; 2 Department of Cardiology, Erasmus University Hospital, Université Libre de Bruxelles, Brussels, Belgium

**Keywords:** Bisoprolol, Exercise aerobic capacity, Chronotropic response

## Abstract

**Background:**

Beta-blockers are increasingly prescribed while the effects of beta-adrenergic receptor blockade on cardio-pulmonary exercise test (CPET)-derived parameters remain under-studied.

**Methods:**

Twenty-one young healthy adults repeated three CPET at the same time with an interval of 7 days between each test. The tests were performed 3 h after a random, double-blind, cross-over single-dose intake of placebo, 2.5 mg or 5.0 mg bisoprolol, a cardio-selective beta1-adrenoreceptor antagonist. Gas exchange, heart rate (HR) and blood pressure (BP) were measured at rest and during cyclo-ergometric incremental CPET.

**Results:**

Maximal workload and VO_2_max were unaffected by the treatment, with maximal respiratory exchange ratio > 1.15 in all tests. A beta-blocker dose-dependent effect reduced resting and maximal BP and HR and the chronotropic response to exercise, evaluated by the HR/VO_2_ slope (placebo: 2.9 ± 0.4 beat/ml/kg; 2.5 mg bisoprolol: 2.4 ± 0.5 beat/ml/kg; 5.0 mg bisoprolol: 2.3 ± 0.4 beat/ml/kg, *p* < 0.001). Ventilation efficiency measured by the VE/VCO_2_ slope and the ventilatory equivalent for CO_2_ at the ventilatory threshold were not affected by beta1-receptor blockade. Post-exercise chronotropic recovery measured after 1 min was enhanced under beta1-blocker (placebo: 26 ± 7 bpm; 2.5 mg bisoprolol: 32 ± 6 bpm; 5.0 mg bisoprolol: 33 ± 6 bpm, *p* < 0.01).

**Conclusion:**

The present results suggest that a single dose of bisoprolol does not affect metabolism, respiratory response and exercise capacity. However, beta-adrenergic blockade dose dependently reduces exercise hemodynamic response by lowering BP and the chronotropic response.

## Introduction

Beta-blockers are a class of pharmacological competitive antagonists of the adrenergic beta-receptors of the sympathetic nervous system allowing for a smoothened endogenous catecholamines response [1]. Beta-blockers are frequently prescribed in coronary artery disease (CAD), congestive heart failure (CHF) [2], cardiac arrhythmias, congenital heart disease, but also for essential tremor [3, 4].

Cardio-pulmonary exercise testing (CPET) in chronic heart disease or in apparently healthy subject with unexplained dyspnea is increasingly promoted [5]. CPET provides a quantitative and qualitative characterization of the oxygen transport system, involving the pulmonary, cardiovascular and muscular oxidative systems [6], with direct measurement of primary outcome variables related to survival and adverse events (Class1-evidence A); aerobic capacity (VO_2_max), minute ventilation/carbon dioxide output slope (VE/VCO_2_) and exercise oscillatory ventilation [6, 7]. Notwithstanding, CPET also assess important secondary outcome variables whose independence in predicting clinical outcome has not yet been consistently reported. These parameters include the oxygen pulse [oxygen consumption (VO_2_) vs heart rate (HR) ratio] used as a surrogate for the left ventricular stroke-volume at exercise, the systolic blood pressure (SBP) or the HR vs VO_2_ slope measuring the chronotropic response to exercise [7, 8]. These secondary outcome variables are additional prognostic predictors of the risk for future adverse events in pathological conditions and in apparently healthy individuals. The chronotropic response to exercise is also widely used for exercise intensity quantification in the context of rehabilitation.

Beta-blockers intake has unequivocally been demonstrated to produce a 20—30% reduction in resting and maximal HR in healthy subjects [9–12]. A compensatory increase in stroke volume through intrinsic cardiac regulation, however, prevents a cardiac output alteration [13–15]. An increase in arteriovenous oxygen content difference (CavO_2_) has also been suggested to prevent a large decrease in VO_2_ during exercise [13–15]. While previous studies in healthy individuals showed trivial influence of beta-blocker intake on VO_2_max and on the ventilatory threshold (VT) level, controversial effects were described on CPET secondary outcome variables [11, 15–17]. The previous conflicting results in heathy subjects may be attributed to the beta-blocker agent, type, posology, intake duration, unknown pathology, fitness level, or additional confounding factors.

We hypothesized that a pharmacological specific beta1-blockade will negatively affect the chronotropic response to exercise and recovery of healthy subjects which could affect the primary and secondary outcome CPET-derived variables. In a prospective double-blind cross-over observational study, we investigated the effects of an acute administration of a high selective beta-blocker (bisoprolol) in normal young subjects on the primary and secondary outcome CPET variables. This is of importance since effects of bisoprolol on primary or secondary outcome CPET parameters may lead to inappropriate interpretation of CPET and misjudgment of mortality risk as those parameters are included in mortality risk calculation algorithms [4].

## Methods

Twenty-six active healthy volunteers recruited on the University Campus participated to the study. The study was approved by the local Institutional Ethics Committee (P2019/504). All participants were recruited from 02/2020 to 04/2020 and gave their written consent. All volunteers were nonsmokers and did not declare any health problems. Their clinical examinations were unremarkable and none of them took any drugs. Their electrocardiograms were normal. Finally, twenty-one subjects were allowed to participate in the study (men) and fully completed the protocol with no drop-out (Table 1).

**Table 1:** Baseline characteristics

	Placebo	2.5 mg	5.0 mg
Age (year)	23 ± 2		
Height (cm)	175 ± 8		
Weight (kg)	70 ± 9		
BMI (kg/m^2^)	22.8 ± 2.4		
HR (b/min)	87 ± 11	73 ± 10***	67 ± 8***^$^
SBP (mmHg)	115 ± 13	104 ± 15 **	97 ± 12***^$^
DBP (mmHg)	71 ± 10	66 ± 6	63 ± 9**
SpO_2_ (%)	99 ± 1	99 ± 1	99 ± 1

BMI: Body Mass Index, HR: Heart Rate; SBP: Systolic Blood Pressure; DBP: Diastolic Blood Pressure; SpO_2_: Pulsed oxygen saturation

**p < 0.01, ***p < 0.001 comparison bisoprolol vs placebo; ^$^*p* < 0.05 comparison 2.5 vs 5.0 mg bisoprolol

Participants underwent a baseline clinical examination followed by a CPET on a cyclo-ergometer on three occasions at the Erasmus Hospital, Brussels with maximum 5 to 7 days of rest between each trial. All three tests were performed at the same time of the day. Subjects were asked to maintain their daily activity levels, maintain their diet habits and abstained from energy or caffeinated drinks for 24 h before testing.

A randomized, double-blind (subject and CPET operator), crossover, placebo-controlled design was conducted, with subjects receiving a sequence of 2.5 mg bisoprolol or 5.0 mg bisoprolol or placebo, strictly 3 h before the beginning of the tests [18]. Hémifumarate of bisoprolol was chosen for its competitive antagonist characteristics conferring a high degree of cardio-selective β-adrenergic receptor blockade with a maximal effect 3 h after intake [18]. 2.5 mg is the minimum initial clinical dose prescribed for heart failure patients but also for patients with atrial fibrillation, as they are prescribed once-daily and do not require dose adjustment in patients with renal impairment. A 5.0 mg dose was also tested to assess the effect of extended β-adrenergic inhibition.

### Clinical Examination

A preliminary screening visit included a full medical history and a physical examination. Subjects were defined as healthy based on the following criteria: (i) no history of acute or chronic disease; (ii) no history of cardiovascular symptoms; (iii) normal blood parameters; (iv) no intake of antioxidant compounds or other medication except for oral contraceptive; and (v) BP and HR within normal limits. Resting BP (by a sphygmomanometer, Medisoft Ergoline 4 M, Hasselt, Belgium), ECG (by electrocardiographic lead; Strässle & CO DT100, Albstadt, Germany) and oxygen saturation (SpO_2_) (measurements of SpO_2_ by pulse oximetry; Nonin 8000S, Tilburg, The Netherlands) were measured during the screening visit and repeated before each experimental session. To avoid excessive bradycardia or hypotension, five subjects with resting HR < 60 bpm or mean BP < 70 mmHg without medication were excluded from the study. In the 21 remaining subjects, no side effects or adverse events were reported before, during and after CPET under placebo or bisoprolol.

### Cardio-Pulmonary Exercise Testing

The cycle ergometer CPET were performed in an upright position as previously reported with a warm-up of 3 min set at 60 W for men and 30 W for women and workload increased by 30 W/min for men and 20 W/min for women until volitional fatigue [19]. The recovery period consisted in pedaling at 60 round per minute at 60 W for men and 30 W for women for 2 min. BP was measured automatically by an electronic sphygmomanometer (Ergoselect 200, Ergoline, Bitz, Germany) at each level of exercise, HR was continuously monitored with a 12-lead electrocardiogram and SpO_2_ were continuously monitored.

During the three exercise testing sessions, breath by breath data of VE, VO_2_ and VCO_2_ were collected and analyzed every 5 s using a metabolic recording system (HypAir, Medisoft, Dinant, Belgium) calibrated with room air and standardized gas before each test. VO_2_max was considered to be achieved when the respiratory exchange ratio (RER) was greater than 1.15. The VT was measured by the V-slope method. The V_E_/VCO_2_ ratio was measured at the VT [6]. The VO_2_/workload slope and the HR/VO_2_ slope were calculated from rest to maximal exercise. The VE/VCO_2_ slope was calculated from rest to the respiratory compensation point as recommended [6]. Maximal voluntary ventilation (MVV) was calculated as forced expiratory volume in one second (FEV1) multiplied by 40 and was considered as the limit of the ventilatory reserve [6]. Maximal O_2_ pulse was found by dividing the VO_2_max by maximal HR. Chronotropic index (CI) was calculated as (HRmax-HRrest)/[(220-age)-HRrest] [20]. HR recovery (HRR) was the difference between maximal HR and HR measured after one and two minutes of recovery.

### Statistics

Results are presented as mean ± standard deviation (SD). The statistical analysis consisted of a repeated measured analysis of variance, with modified t tests (Bonferroni) to compare different β-blockers doses when the F-ratio of the analysis of variance reached a *p* < 0.05 critical value. Statistical analysis was performed using Statistica (Statistica version 10, StatSoft, Tulsa, USA).

## Results

### Rest

The effect of placebo or different bisoprolol doses intake on resting hemodynamic parameters is shown in Table 1. 2.5 mg bisoprolol intake decreased resting HR by 14 bpm and SBP by 11 mmHg with no statistical influence on diastolic BP (DBP), while 5.0 mg bisoprolol reduced resting HR by 20 bpm, SBP by 18 mmHg and DBP by 8 mmHg. SpO_2_ remained unaffected.

### Ventilatory Threshold

As exposed in Table 2, the VT level did not differ between the three pharmacological conditions with unchanged workload, absolute VO_2_ (L/min) or VO_2_ relative to VO_2_max (% VO_2_max) and the VE/VCO_2_ ratio. However, the intake of bisoprolol dose dependently reduced HR, SBP and DBP at this submaximal exercise level.

**Table 2:** Cardio-pulmonary exercise variables after bisoprolol vs placebo

	Placebo	2.5 mg	5.0 mg
*Ventilatory threshold*			
HR (b/min)	143 ± 17	119 ± 12***	111 ± 12***^$$^
SBP (mmHg)	151 ± 16	130 ± 21 ***	117 ± 14 ***^$^
DBP (mmHg)	73 ± 10	68 ± 8	64 ± 8**
VO_2_ (L/min)	1.8 ± 0.5	1.7 ± 0.4	1.6 ± 0.4
VE/VCO_2 ratio_	28 ± 3	28 ± 4	28 ± 4
*Maximal exercise*			
RER	1.29 ± 0.09	1.27 ± 0.07	1.30 ± 0.08
HR (b/min)	183 ± 8	160 ± 11***	151 ± 12***^$$^
Chronotropic Index	0.87 ± 0.06	0.71 ± 0.08***	0.65 ± 0.08***^$$^
SBP (mmHg)	196 ± 20	179 ± 23 **	160 ± 22 ***^$$^
DBP (mmHg)	81 ± 13	78 ± 14	73 ± 11*
Workload (Watt)	246 ± 48	245 ± 52	238 ± 42
VO_2_ (L/min)	2.7 ± 0.5	2.7 ± 0.5	2.6 ± 0.5
VO_2_ (mL/kg/min)	39 ± 5	39 ± 5	37 ± 5
O_2 Pulse_ (mL/b/min)	15.0 ± 3.2	17.1 ± 3.7*	17.3 ± 3.7*
VE (L/min)	114 ± 16	105 ± 16	104 ± 21
VE/MVV (%)	78 ± 9	74 ± 9	74 ± 12
SpO_2_ (%)	*98* ± *2*	*98* ± *2*	*98* ± *2*
*Slopes*			
VE/VCO_2_	27 ± 3	26 ± 3	26 ± 4
HR/VO_2_ (b/mL/kg)	2.9 ± 0.4	2.4 ± 0.5***	2.3 ± 0.4***
VO_2_/W (mL/min/watt)	10 ± 1	10 ± 1	10 ± 1
*Recovery*			
HRR 1' (b/min)	26 ± 7	32 ± 6**	33 ± 6**
HRR 2' (b/min)	45 ± 7	48 ± 6	46 ± 7

RER: Respiratory Exchange Ratio; HR: Heart Rate; SBP: Systolic Blood Pressure; DBP: Diastolic Blood Pressure; VO2: Oxygen consumption; VE: Ventilation; MVV: Maximal Voluntary Ventilation; HRR: Heart Rate Recovery

*p < 0.05, **p < 0.01, ***p < 0.001 both measurements vs placebo; ^$$^*p* < 0.01 comparison 2.5 vs 5.0 mg bisoprolol

### Maximal Exercise

All exercise tests reached a metabolic maximality with end exercise RER systematically exceeding 1.15 (Table 2).

**Fig. 1:**
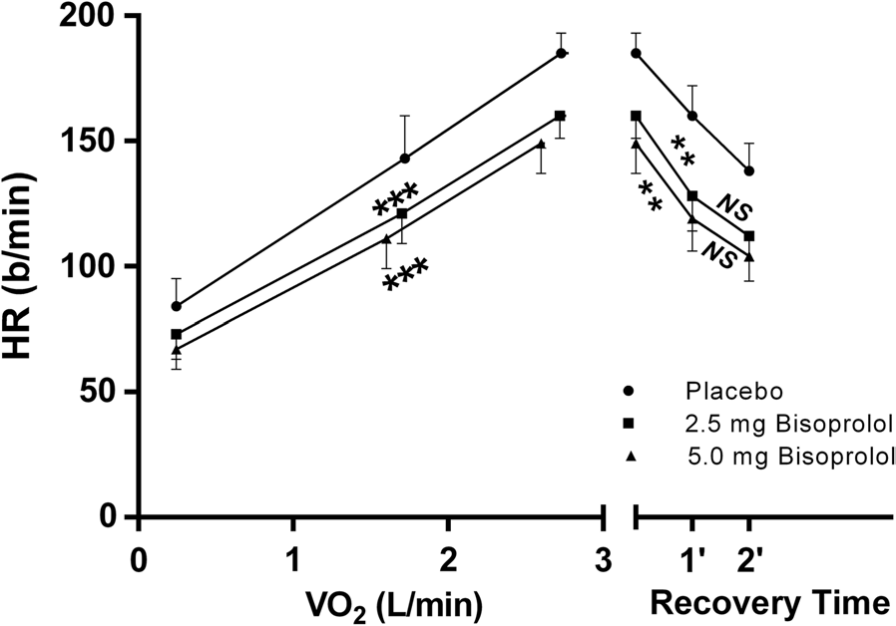
Chronotropic response during and after an incremental exercise. Heart rate (HR) measurements were reported at rest, at the ventilatory threshold, at maximal exercise, after 1 and 2 min of recovery. The slope of the HR vs oxygen consumption (VO_2_) measured during CPET is negatively impacted by the intake of 2.5 or 5.0 mg of bisoprolol (dose response). Bisoprolol also increased the HR recovery after 1 min (difference between maximal HR and HR measured 1 min after the end of maximal exercise), but not after 2 min under β1-blockade compared to placebo. No difference between 2.5 and 5.0 mg of acute bisoprolol intake were observable

**Fig. 2:**
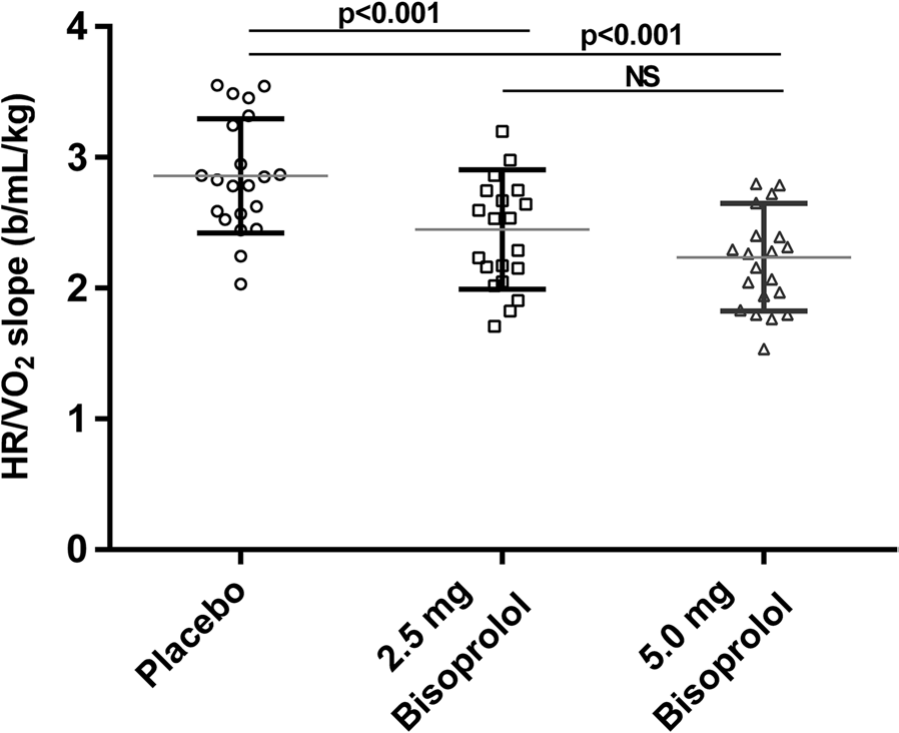
Individual CPET derived HR/VO_2_ slopes measured in healthy subjects under placebo, 2.5 mg bisoprolol or 5.0 mg of bisoprolol. Mean values are represented by large horizontal bars and standard deviation by vertical bars in each condition. B1 adrenoreceptors blockade negatively affected the HR/VO_2_ slope

**Fig. 3:**
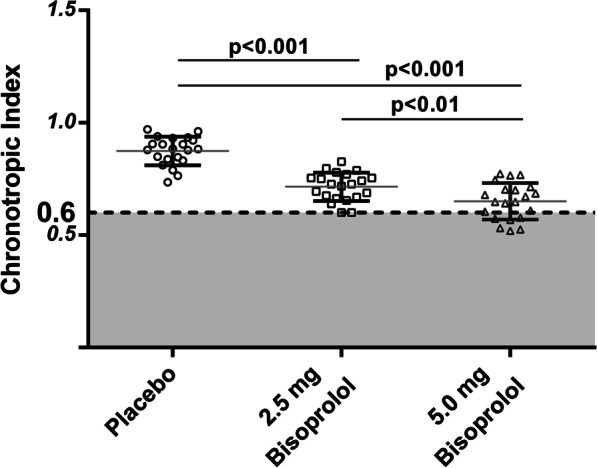
Calculated chronotropic index (CI) in healthy subjects under placebo, 2.5 mg bisoprolol or 5.0 mg of bisoprolol. Mean values are represented by large horizontal bars and standard deviation by vertical bars in each condition. 7 subjects out of 21 (33%) under 5.0 mg of bisoprolol are included in the chronotropic incompetence zone (grey zone) situated below a chronotropic index of 0.6

Maximal SBP, DBP and HR were decreased by β1-receptor blockade as compared to the placebo condition with an increased effect after 5.0 mg of bisoprolol compared to 2.5 mg (Fig. 1). HR/VO_2_ slope and CI also decreased with the use of bisoprolol (2.5 and 5.0 mg) (Figs. 2 and 3). 2.5 mg or 5.0 mg bisoprolol increased maximal O_2_ pulse (Table 2). Bisoprolol had no effect on maximal VE, workload, VO_2_, SpO_2_ and on the VE/VCO_2_ or VO_2_/W slopes.

### End Exercise Recovery

β1-blockade had no effect on gas exchange or ventilatory parameters during the recovery period (not shown). However, enhanced chronotropic recovery was observable with the use of bisoprolol (2.5 and 5.0 mg) within the first minute post-exercise but returned to normal at 2 min of recovery (Table 2).

## Discussion

β-blocker is a commonly used drug class whose influence remains under-considered in the interpretation of CPET. In the present study, acute inhibition of cardio-specific β-adrenoreceptors had no effect on aerobic capacity or ventilatory response during exercise in healthy subjects. Preserved VO_2_max was possible since the β-blockers negative chronotropic and inotropic effects, assessed by lower resting and maximal HR and SBP with a 18% reduction in the HR/VO_2_ slope, was counter-balanced by increased O_2_ pulse probably through intrinsic cardiac and/or peripheral adaptative mechanisms. The present result also revealed a 23% increase in the early chronotropic recovery within the first end-exercise minute.

### Beta-Blockade

The bisoprolol dose used in the present study respected the recommendations when initiating a treatment titrated to effect [21]. How much body-weight titration would have influenced the present results is unknown. After 2.5 mg bisoprolol, 97% of the subjects showed a clear chronotropic inhibition, > 10 bpm, indicating effective starting dose.

With 5 mg of bisoprolol, all subjects decrease HR at rest with no bradycardiac adverse events reported. 76% of the subjects reached the minimal tolerated HR (70 bpm) suggesting achievement of the target dose. It remains unknown if higher doses would have been more efficient the 24% other subjects.

### Aerobic Capacity

Previous studies reported either an unchanged or a 5–7% VO_2_max alteration after 1 or 2 weeks of β-blocker intake [16, 17]. No altered VO_2_max was observed in the present study in accordance with other studies on healthy young subjects [22, 23], aortic aneurysm [14], or hypertension patients [24]. This general observation made in healthy or pathological conditions suggests that the negative inotropic and chronotropic effects of β-blocker promote multiple compensatory-dependent mechanisms such as; mechanical/intrinsic cardiac adaptation and/or according to Fick’s principle, enhanced O_2_ extraction in exercising muscles, etc. [15].

### Chronotropic Response to Exercise

HR response to exercise is multi-factorial and depends on autonomic outflows (central command), reflex responses to skeletal muscle activation (exercise pressor reflex), hemodynamic changes, sinus node function, parasympathetic withdrawal and β-adrenoreceptor responsiveness [20]. At the onset of exercise, chronotropic response mainly depends on para-sympathetic drive reduction. Further exercising allows the catecholamines to stimulate nodal cells and cardiomyocytes β1-receptors which will in turn modify the membrane permeability to K^+^ and Ca^++^ resulting in increased cardiomyocytes excitability, frequency of excitation, impulse conduction and relaxation speed [20]. Pharmacological inhibition of the β1-receptors will therefore attenuate all those cardiac adrenergic responses during exercise and might also participate to the smoothened SBP response at exercise [20].

### Chronotropic Index

Chronotropic incompetence, defined as the inability of the HR to increase during exercise, is diagnosed using the chronotropic index, the HR/VO_2_ slope or when the measured maximal HR does not reach 80% of the predicted maximal HR [20, 25].

Numerous studies showed an altered chronotropic index under chronic and acute β-blockers intakes [25–27]. However, the chronotropic index has also been shown to be inversely correlated to mortality in healthy men [28], congenital heart disease [29]. Also in patients with HF treated with β-blockers, an index below 0.6 increased mortality of + 17% at 24 months [30]. As illustrated in Fig. 3, 7 healthy subjects out of 21 (33%) showed a chronotropic index < 0.6 after 5.0 mg of bisoprolol intake. Hence in some circumstances, the chronotropic index might be more affected by the treatment than by the disease itself.

### HR/VO_2_ Slope

The HR vs metabolism relationship, namely the HR/VO_2_ or HR/MET slopes, also evaluates the chronotropic response to exercise with the advantage to be physical fitness level independent. Normal values of HR/VO_2_ are between 3 to 4 b/ml/kg in healthy sedentary subjects [6] and is known to be influenced by age, sex, physical fitness or altitude [23, 31, 32]. The HR/VO_2_ slope is increased in cardiac affections such as Fontant patients (not on beta-blockers) or atrial septal defect and reduced in patients with heart failure with preserved ejection fraction treated by beta-blockers [33–35]. The presently healthy subjects exhibited a decreased HR/VO_2_ slope after bisoprolol intake reaching the lower limit of normal independently of the prescribed dose (Fig. 2). Our study highlights that a drug-related reduction in the HR/VO_2_ slope (average decrease of 0.5 b/ml/kg) has to be taken into account during CPET analysis, when beta-blockers are prescribed, at least acutely.

### Chronotropic Post-Exercise Kinetics

HRR is often reported as an indirect estimation of cardio-vascular fitness. Indeed, HRR is enhanced in endurance athletes as compared to resistive training athletes [20]. Conversely, a delayed HRR, particularly when < 12 bpm decrease is observed within the first minute after maximal exercise, is associated with increased all-cause mortality in asymptomatic subjects and in pathological populations such as HF, chronic obstructive pulmonary disease or interstitial lung disease [6, 8, 36, 37]. Indeed, this early recovery phase is highly dependent on the reactivation of the parasympathetic tone, while later (slow recovery phase), HRR is influenced by the reduction of sympathetic activity and non-autonomic factors (α-adrenergic tone, atrial stretch or central temperature changes) [38].

The present increased HRR at 1 min post-exercise suggest that acute β1-adrenergic blockade may allow for an enhanced cardiac vagal reactivation. However, the involvement of non-autonomic mechanisms cannot be excluded [38].

### Oxygen Pulse

IN the present study, maximal O_2_ pulse, a composite index reflecting maximal stroke volume and end-exercise peripheral O_2_ extraction, was increased by the intake of β1-blocker independently of the drug dose. As end-exercise peripheral O_2_ extraction has been shown to be unaltered following B1-selective blockade [12], increased maximal O_2_ pulse may reflect a decreased systemic resistance or left ventricular afterload and an intrinsic cardiac adaptation after β-adrenergic receptor blockade, resulting from the Frank-Starling mechanism when ventricular filling time is increased.

β1-selective blockade is also known to preserve the β2-receptor induced muscular vasodilation during exercise with one study reporting a 4% increase in end exercise CavO_2_ of CAD patients [13]. This suggests that this peripheral adaptation may partially contribute to the increased maximal O_2_ pulse and the VO_2_max preservation under β1-blockade.

### Gas Exchange and Chemosensibility

The present result showed no influence of bisoprolol intake on the ventilatory response to exercise, preserving maximal ventilation but also ventilatory efficiency evaluated by the VE/VCO_2_ ratio at VT or the VE/VCO_2_ slope. This suggests little or no interference of β1-adrenergic effects on central or peripheral chemo-receptors or muscle metabo-receptors during exercise [39]. However, Beloka et al. previously showed smoothened VE/VCO_2_ slopes after chronic intake of bisoprolol in healthy subjects [17]. Because this was not associated with detectable changes in the sympathetic nervous system tone, metabosensitivity or chemosensitivity, the authors attributed the lower VE/VCO_2_ slopes to underlying hemodynamic mechanisms. Other studies showing lower hyperventilation response under β-blockers were realized under un-specific β blockade, suggesting an involvement of β2-adrenoreceptors rather than β1-receptors in the ventilatory response to exercise [39].

### Clinical Relevance

From a clinically point of view, the present study highlights a potential risk of misinterpretation of CPET in subjects on beta-blockers. Firstly, it is interesting to note that VO_2_max is not or hardly affected by beta-blocker intake. Secondly, the maximal O_2_pulse value will be overestimated due to the negative chronotropic effect suggesting a cautious and contextualized interpretation. Thirdly, the chronotropic response to exercise (assessed by the chronotropic index or HR/VO_2_ slope) is reduced by an average of 0.5 b/mL/kg and should be taken into account in the interpretation, especially when the value approaches the limit of chronotropic incompetence (< 3 b/mL/kg). Finally, the pharmacologically-induced HRR acceleration with bisoprolol should be included in the interpretation of the recovery phase.

### Limitation of the Study

There are several limitations in the present study that could have affected the results or conclusions. All tested subjects were healthy active young adults. The extrapolation of the present results to older subjects or to patient with cardiac diseases remains therefore uncertain. It is also important to underly that five subjects were precautionary excluded for high reactivity to bisoprolol with bradycardia and hypotension. This thus constitutes an inclusion bias in the present study.

Moreover, the present observed effects are the consequence of an acute β-blocker dose intake and it remains uncertain if more β1-receptors would be inhibited with a chronic β-blocker treatment or higher doses and if it would influence the present results.

Also, young men and women were recruited in the present study sample, but sex influence on chronotropic response under beta-blockade remains unknown. Although no sex differences appeared after an inter-sex comparison (not shown), a sex influence cannot be excluded in older or pathological populations.

## Conclusion

In conclusion, even if acute β-blocker intake has little or no influence on aerobic capacity, it however affects the chronotropic response to exercise, by lowering the chronotropic index or the HR/VO_2_ slope and increasing the HR recovery during the first min post-exercise.

This should be taken into consideration during CPET interpretation for prognostic establishments, follow-up but also for HR-based exercise titration. As a general recommendation, this study and previous evidence suggest CPET should be performed only under chronic stabilized beta-blockers therapy in order to facilitate the interpretation of the results.

## Data Availability

The datasets generated during and/or analyzed during the current study are available from the corresponding author on reasonable request.

## Abbreviations

BMI: Body mass index
CAD: Coronary artery disease
CavO_2_: Arteriovenous oxygen content difference
CHF: Congestive heart failure
CI: Chronotropic index
CPET: Cardio-pulmonary exercise testing
DBP: Diastolic blood pressure
HR: Heart rate
HRR: Heart rate recovery
MVV: Maximal voluntary ventilation
RER: Respiratory exchange ratio
SBP: Systolic blood pressure
SD: Standard deviation
SpO_2_: Pulsed oxygen saturation
VE: Ventilation
VO_2_: Oxygen consumption
VT: Ventilatory threshold
